# WEE1 epigenetically modulates 5-hmC levels by pY37-H2B dependent regulation of *IDH2* gene expression

**DOI:** 10.18632/oncotarget.22374

**Published:** 2017-11-10

**Authors:** Nupam P. Mahajan, Pavani Malla, Shambhavi Bhagwat, Vasundhara Sharma, Amod Sarnaik, Jongphil Kim, Shari Pilon-Thomas, Jeffery Weber, Kiran Mahajan

**Affiliations:** ^1^ Department of Drug Discovery, Moffitt Cancer Center, Tampa, FL 33612, USA; ^2^ Department of Tumor Biology, Moffitt Cancer Center, Tampa, FL 33612, USA; ^3^ Department of Cutaneous Oncology, Moffitt Cancer Center, Tampa, FL 33612, USA; ^4^ Department of Biostatistics and Bioinformatics, Moffitt Cancer Center, Tampa, FL 33612, USA; ^5^ Department of Immunology, Moffitt Cancer Center, Tampa, FL 33612, USA; ^6^ Department of Oncological Sciences, University of South Florida, Tampa, FL 33612, USA; ^7^ Laura and Isaac Perlmutter Cancer Center, NYU Langone Medical Center, New York, NY 10016, USA

**Keywords:** WEE1, histone H2B, tyrosine phosphorylation, IDH2, tyrosine kinase

## Abstract

Epigenetic signaling networks dynamically regulate gene expression to maintain cellular homeostasis. Previously, we uncovered that WEE1 phosphorylates histone H2B at tyrosine 37 (pY37-H2B) to negatively regulate global histone transcriptional output. Although pY37-H2B is readily detected in cancer cells, its functional role in pathogenesis is not known. Herein, we show that WEE1 deposits the pY37-H2B marks within the tumor suppressor gene, isocitrate dehydrogenase 2 (*IDH2*), to repress transcription in multiple cancer cells, including glioblastoma multiforme (GBMs), melanoma and prostate cancer. Consistently, GBMs and primary melanoma tumors that display elevated WEE1 mRNA expression exhibit significant down regulation of the *IDH2* gene transcription. IDH2 catalyzes the oxidative decarboxylation of isocitrate to α-ketoglutarate (α-KG), an essential cofactor for the TET family of 5-methylcytosine (5mC) hydroxylases that convert 5-mC to 5-hydroxymethylcytosine (5-hmC). Significantly, the WEE1 inhibitor AZD1775 not only abrogated the suppressive H2B Y37-phosphorylation and upregulated *IDH2* mRNA levels but also effectively reversed the ‘loss of 5-hmC’ phenotype in melanomas, GBMs and prostate cancer cells, as well as melanoma xenograft tumors. These data indicate that the epigenetic repression of *IDH2* by WEE1/pY37-H2B circuit may be a hitherto unknown mechanism of global 5-hmC loss observed in human malignancies.

## INTRODUCTION

The precise execution of epigenetic signaling events, which includes both addition and removal of epigenetic marks in a temporally regulated manner, is vital to preserve the chromatin landscape and maintain cellular homeostasis. Deregulated epigenetic signaling networks profoundly alter the epigenome that is linked to cancer and developmental diseases [1–6]. It has become evident that behind every histone or DNA modification, commonly referred to as epigenetic changes, there are a number of regulators that work in a highly orchestrated manner to govern the transcriptional landscape and determine cell fate. Modification of tyrosine residues in proteins functions as a precise on-off switch and has acquired considerable significance particularly in light of the evidence of histones being modified directly by disparate kinases in recent years [7–14]. Of the 6 known histone tyrosine phosphorylation events, WEE1 directly phosphorylates the histone H2B at tyrosine 37 (pY37-H2B) to negatively regulate global histone transcriptional output in a temporally regulated manner during each cell cycle [7, 13]. The histone transcriptional repressor, HIRA ‛a reader’ of the pY37-H2B epigenetic marks, recognizes these marks, displaces a transcriptional coactivator to suppress global histone mRNA synthesis at the end of DNA synthesis. Interestingly, WEE1 kinase is reported to be aberrantly expressed in malignant melanomas, GBMs and triple-negative and luminal breast cancers [15–19]. The WEE1 inhibitor, AZD1775, also known as MK-1775 [20, 21] potently radiosensitizes human tumor cells [22, 23], suggesting that these tumors rely on WEE1 kinase activity for chromatin integrity. To better understand how cancer cells may benefit from this newly discovered WEE1 epigenetic activity, we mined our pY37-H2B chromatin immunoprecipitation (ChIP)-sequencing data for genes associated with oncogenesis; it revealed that pY37-H2B marks were deposited at an important tumor suppressor gene-the isocitrate dehydrogenase 2 (*IDH2*).

The isocitrate dehydrogenases, IDH1 and IDH2, catalyze the oxidative decarboxylation of isocitrate to α-ketoglutarate (α-KG), a critical regulator of cell metabolism [24, 25]. About 60 dioxygenases expressed in mammalian cells utilize α-KG as an essential cofactor in the oxidation reaction, including the recently discovered TET family of 5-methylcytosine (5mC) hydroxylases that convert 5-mC to 5-hydroxymethylcytosine (5-hmC). A significant decrease or loss of 5-hmC has been identified as a recurrent epigenetic phenomenon in a large number of human malignancies, including melanomas, glioblastoma multiforme (GBM), clear cell renal cell carcinoma, urothelial cell carcinoma, breast, liver, lung, pancreatic and prostate cancers [26–30]. The significance of 5-hmC loss specifically in cancer cells became further evident when reintroduction of the 5-hmC by recombinant IDH2 or TET2, suppressed tumor growth and increased tumor-free survival in the melanoma mouse models [26]. Taken together, it has become apparent that the global loss of 5-hmC is a significant occurrence characteristic of malignancy [31, 32], opening the selective restoration of 5-hmC levels as a new therapeutic option with small molecule inhibitors.

While the down-regulation of *IDH2* is linked to the loss of 5-hmC in human tumors, the epigenetic mechanisms by which *IDH2* gene expression is repressed in human tumors is not known. Moreover although mutations in the *IDH1/IDH2* genes underlie global 5hmC loss [24], these do not account for all the cases observed [27, 32]. Serendipitously, we uncovered that deposition of pY37-H2B epigenetic marks in the *IDH2* gene directly correlated with reduced gene expression. Further, this decrease in *IDH2* gene expression correlated with reduced 5-hmC levels, suggesting that the overexpression of WEE1 in cancer cells may suppress the expression of *IDH2*, leading to decreased 5-hmC levels. Further, increased WEE1 expression in glioblastomas and melanomas positively correlated with decreased *IDH2* expression levels. Conversely, inhibition of WEE1 kinase activity by the selective inhibitor AZD1775, erased the pY37-H2B repressive marks leading to a marked increase in the levels of *IDH2*, thus reversing the ‛loss of 5-hmC’ phenotype. Overall, our data reveal a novel mode of WEE1 mediated *IDH2* downregulation as a recurrent epigenetic alteration that may be operational in melanomas, GBMs and prostate cancers.

## RESULTS

### H2B Y37-phosphorylation is a hallmark of multiple cancer cell lines

**Figure 1:**
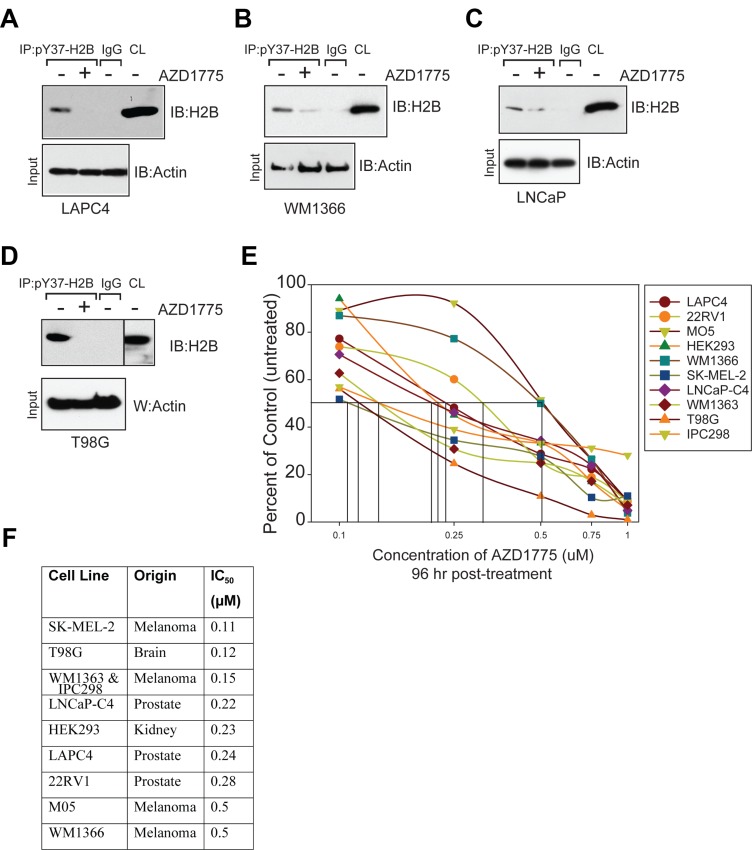
WEE1 inhibition suppresses pY37-H2B expression (**A**–**D**) Prostate cancer (LAPC4 and LNCaP), melanoma (WM1366) and brain cancer (T98G) derived cells were treated with AZD1775 (2 µM, 24 hr) and lysates were immunoprecipitated with pY37-H2B antibodies, followed by immunoblotting with pan-H2B antibodies (top panels). CL indicates the crude lysates. Lower panel indicates immunoblotting of lysates with actin antibodies. (**E**) Cell viability assay. Melanoma, prostate and brain cancer cell lines were treated with AZD1775 for 96 hr and live/dead population was assessed by Trypan blue staining. Data represent mean ± standard error of the mean or s.e.m. (*n* = 3). (**F**) Table indicates IC_50_ values based on cell proliferation analysis in AZD1775 treated cells in comparison to untreated cells shown in Figure 1E.

To interrogate a role for WEE/pY37-H2B epigenetic regulation in promoting human malignancies, melanoma (WM1366), prostate (LNCaP and LAPC4) and brain (T98G) cancer cell lines were first examined. H2B Y37-phosphorylation was a significant feature in several cancer cell lines tested (Figure 1A–1D, top panels). To determine whether pY37-H2B deposition can be reversed, cancer cells of multiple origins were treated with AZD1775, a WEE1 selective inhibitor. H2B Y37-phosphorylation was inhibited in all cancer models following treatment with AZD1775 (Figure 1A–1D, top panels).

To determine the consequence of suppression of H2B Y37-phosphorylation, various melanoma (SK-MEL-2, WM1363, MO5, IPC298 and WM1366), prostate (LNCaP-C4 and LAPC4), GBM (T98G) and HEK293 cells were treated with AZD1775 and the effect on cell proliferation was assessed (Figure 1E). The cells lines tested were highly sensitive to AZD1775-IC_50_ values ranged from 100–500 nM (Figure 1F). An increased accumulation of cells in S phase was observed as a result of treatment with AZD1775 (Supplementary Figure 1). Combined, these data demonstrate that WEE1 mediated H2B Y37-phosphorylation is a recurrent pro-proliferative epigenetic modification in multiple proliferating cancer cells that can be specifically targeted with the WEE1 inhibitor, AZD1775.

### WEE1 marks the *IDH2* gene locus with H2B Y37-phosphorylation

**Figure 2:**
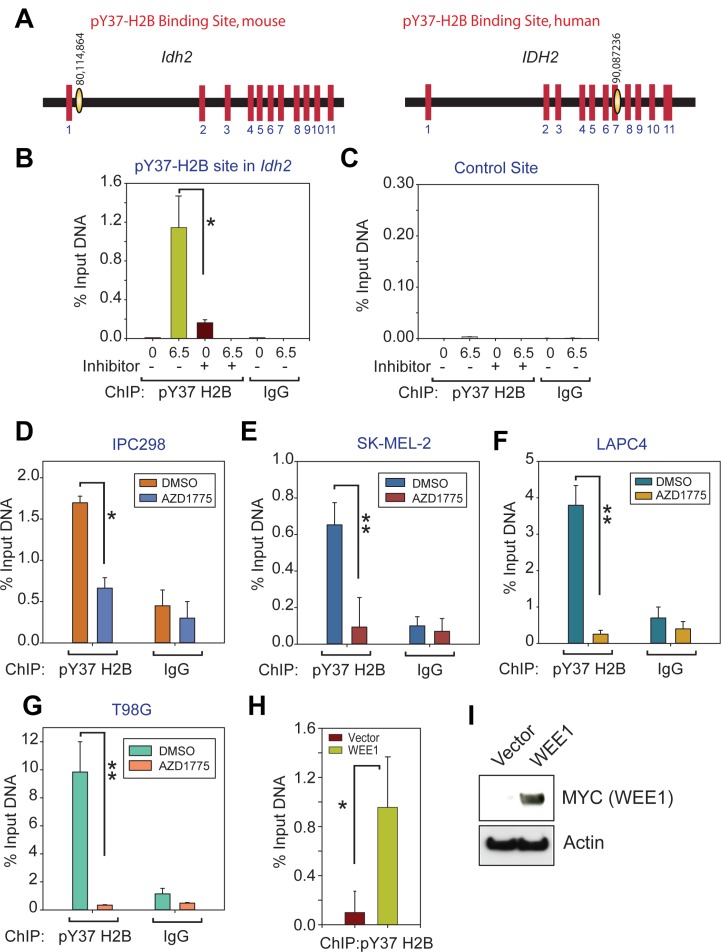
Deposition of WEE1 mediated pY37-H2B epigenetic marks within the *IDH2* gene locus (**A**) Deposition of pY37-H2B epigenetic marks in the mouse *Idh2* and human *IDH2* locus are shown. The red boxes are the exons and yellow ovals are the sites of pY37-H2B deposition. (**B**) ChIP with pY37-H2B and IgG antibodies, followed by qPCR with primers corresponding to the pY37-H2B binding site of mouse embryo fibroblasts (MEFs). Data represent mean ± s.e.m. (*n* = 3. ^*^p < 0.01. (**C**) ChIP with pY37-H2B and IgG antibodies, followed by qPCR with primers corresponding to the control site of MEFs untreated or treated with AZD1775. Data represent mean ± s.e.m. (*n* = 3). (**D**–**G**) ChIP with pY37-H2B and IgG antibodies, followed by qPCR with primers corresponding to the pY37-H2B binding site of human *IDH2* in IPC298, SK-MEL-2, LAPC4 and T98G cells. Data represent mean ± s.e.m. (*n* = 3). ^*^p < 0.05, ^**^p < 0.01. (**H**) ChIP-qPCR of chromatin isolated from HEK293 cells transfected with the vector and WEE1 expressing constructs. Data represent mean ± s.e.m. (*n* = 3). ^*^p < 0.01. (**I**) Immunoblotting of HEK293 lysates transfected with MYC-tagged WEE1 using anti-MYC antibodies.

Earlier, we demonstrated that the WEE1 epigenetic activity is temporally regulated wherein the pY37-H2B epigenetic marks are specifically deposited at the end of S phase during each cell cycle [7]. We interrogated how H2B Y37-phosphorylation influences the transcriptional landscape to regulate cancer cell survival. Towards this goal, the genomic distribution of pY37-H2B epigenetic marks in normal cells, mouse embryo fibroblasts (MEFs) were examined. To identify the genes that are specifically regulated by WEE1, MEFs were synchronized by double thymidine and were harvested 6.30 hours post-release in fresh media. Based on the cell cycle analysis that we have performed earlier, the 6.30 hours post-release time point represents the late S phase, prior to entry into G2/M phase of cell cycle [7]. ChIP-sequencing was performed using pY37-H2B antibodies as described earlier [7]. It revealed that pY37-H2B marks are deposited not only upstream of *Hist1* locus, where large number of histone coding genes are located, but also in many other genes [7]. We performed detailed analysis of the pY37-H2B ChIP- sequencing data and uncovered that these epigenetic marks are deposited in the mouse *Idh2* intron1–2 (80,114,198 to 80,114,864 nt) (Figure 2A and Supplementary Table 1).

To validate the deposition of the pY37-H2B epigenetic marks within the mouse *Idh2* coding region, MEFs were synchronized by double thymidine and were harvested immediately (0 hours post-release) or 6.30 hours post-release in fresh media, which corresponds to G1/S and late S phase of cell cycle, respectively. Directed ChIP-qPCR was performed, which confirmed deposition within the *Idh2* gene at 6.30 hours but not at 0 hours, when WEE1 epigenetic activity is undetectable (Figure 2B). Further, treatment of cells with AZD1775 abolished the deposition of pY37-H2B epigenetic marks at *Idh2* locus. As a control, we used primers corresponding to the ‛Gene desert’ on chromosome 6, where pY37-H2B marks were not detected (control site, Figure 2C). To identify the corresponding region in human *IDH2* gene, we performed chromatin immunoprecipitation (ChIP) with pY37-H2B antibodies followed by PCR with 6 sets of primers spanning *IDH2* gene (90,083,978 to 90,102,554 nt). This led to the identification of the putative pY37-H2B-binding site in human cells (90,087,067 -90,087,236) located in exon 7 and part of intron 7 (Figure 2A and Supplementary Table 1). The primer sequences for human *IDH2* and mouse *Idh2* CHIP are shown in the methods section.

To examine the presence of pY37-H2B marks within the human *IDH2* gene locus, melanoma derived cell lines IPC298 and SK-MEL2, prostate cancer derived cell lines- LAPC4 and GBM derived cell line T98G were treated with WEE1 kinase inhibitor AZD1775 and sheared chromatin was prepared. ChIP was performed using the pY37-H2B antibody, followed by qPCR using primers corresponding to the pY37-H2B-binding site in the human *IDH2* gene. A significant decrease in the pY37-H2B deposition in the *IDH2* gene was observed upon WEE1 inhibition (Figure 2D–2G). To further validate a direct role for WEE1 in the deposition of the pY37-H2B epigenetic marks in human *IDH2*, ChIP was performed for the HEK293 cells that were transfected with the WEE1 kinase, followed by qPCR for pY37-H2B binding sites with the selected primer pairs. A significant increase in the pY37-H2B deposition in the *IDH2* gene was observed in WEE1 overexpressing cells (Figure 2H and 2I). Taken together these data indicate the ability of WEE1 to epigenetically mark *IDH2* locus is conserved in both mouse and human cells.

### WEE1/pY37-H2B epigenetic axis suppresses *IDH2* gene transcription

**Figure 3:**
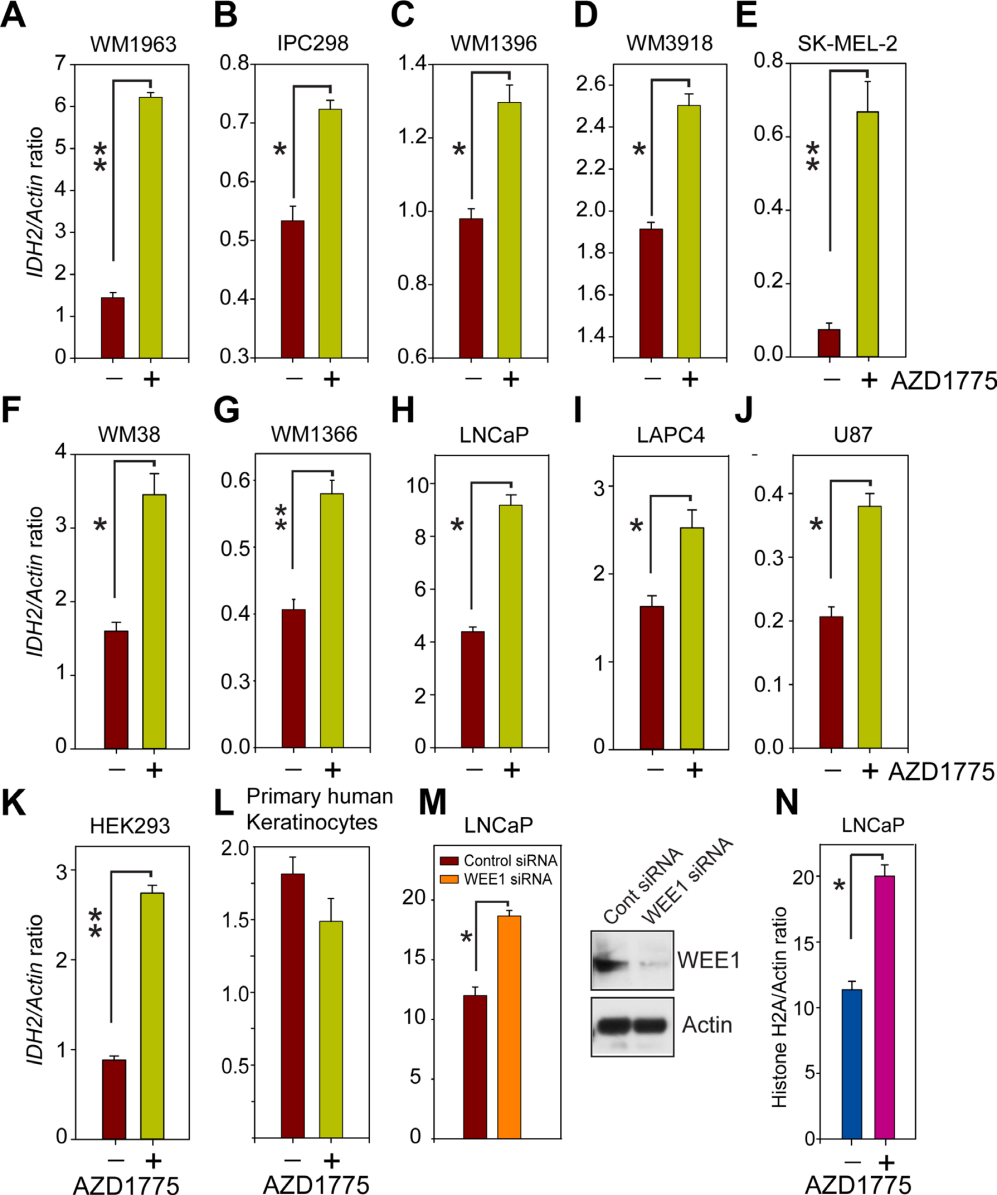
Inhibition of WEE1 kinase activity enhances *IDH2* mRNA expression (**A**–**L**) Quantitative reverse transcription polymerase chain reaction (qRT–PCR) analysis of *IDH2* mRNA expression in indicated cells treated with AZD1775 (1 µM, 24 hr). Data represent mean ± s.e.m. (*n* = 3). ^*^p < 0.05, ^**^p < 0.01. (**M**) qRT–PCR analysis of *IDH2* in LNCaP cells transfected with WEE1 siRNA. Data represent mean ± s.e.m. (*n* = 3). ^*^p < 0.05. Loss of WEE1 expression is confirmed by immunoblotting. (**N**) qRT–PCR analysis of histone H2A mRNA in LNCaP cells treated with WEE1 inhibitor, AZD1775. Data represent mean ± s.e.m. (*n* = 3). ^*^p < 0.05.

To investigate whether WEE1/pY37-H2B signaling plays a direct role in regulating *IDH2* gene transcription, various melanoma (WM1963, IPC298, WM1396, WM3918, SK-MEL-2, WM38 and WM1366), prostate (LNCaP and LAPC4), GBM (U87) and HEK293 cells were treated with WEE1-specific inhibitor, AZD1775. RNA was prepared followed by real time PCR with *IDH2*-specific primers. A significant increase in *IDH2* transcript levels was observed upon WEE1 inhibition (Figure 3A–3K). As a control, we utilized a primary human keratinocytes; interestingly, *IDH2* mRNA levels remained unchanged in these non-cancerous cells despite AZD1775 treatment (Figure 3L), suggesting a cancer specific regulation of IDH2 by pY37-H2B/WEE1 signaling axis.

To examine whether increased IDH2 mRNA levels upon AZD1775 treatment is not due to an ‛off-target effect’ of the inhibitor, LNCaP cells were transfected with WEE1-specific siRNA and control siRNA. A significant upregulation of *IDH2* mRNA levels was observed upon WEE1 knockdown in LNCaP cells (Figure 3M). Earlier we demonstrated that WEE1 negatively regulates global histone output [7]. Consistent with this notion, WEE1 inhibition resulted in a significant increase in histone H2A mRNA levels (Figure 3N). Taken collectively, these data indicate that the deposition of the pY37-H2B epigenetic marks by WEE1 has a repressive effect on *IDH2* gene regulation.

**Figure 4:**
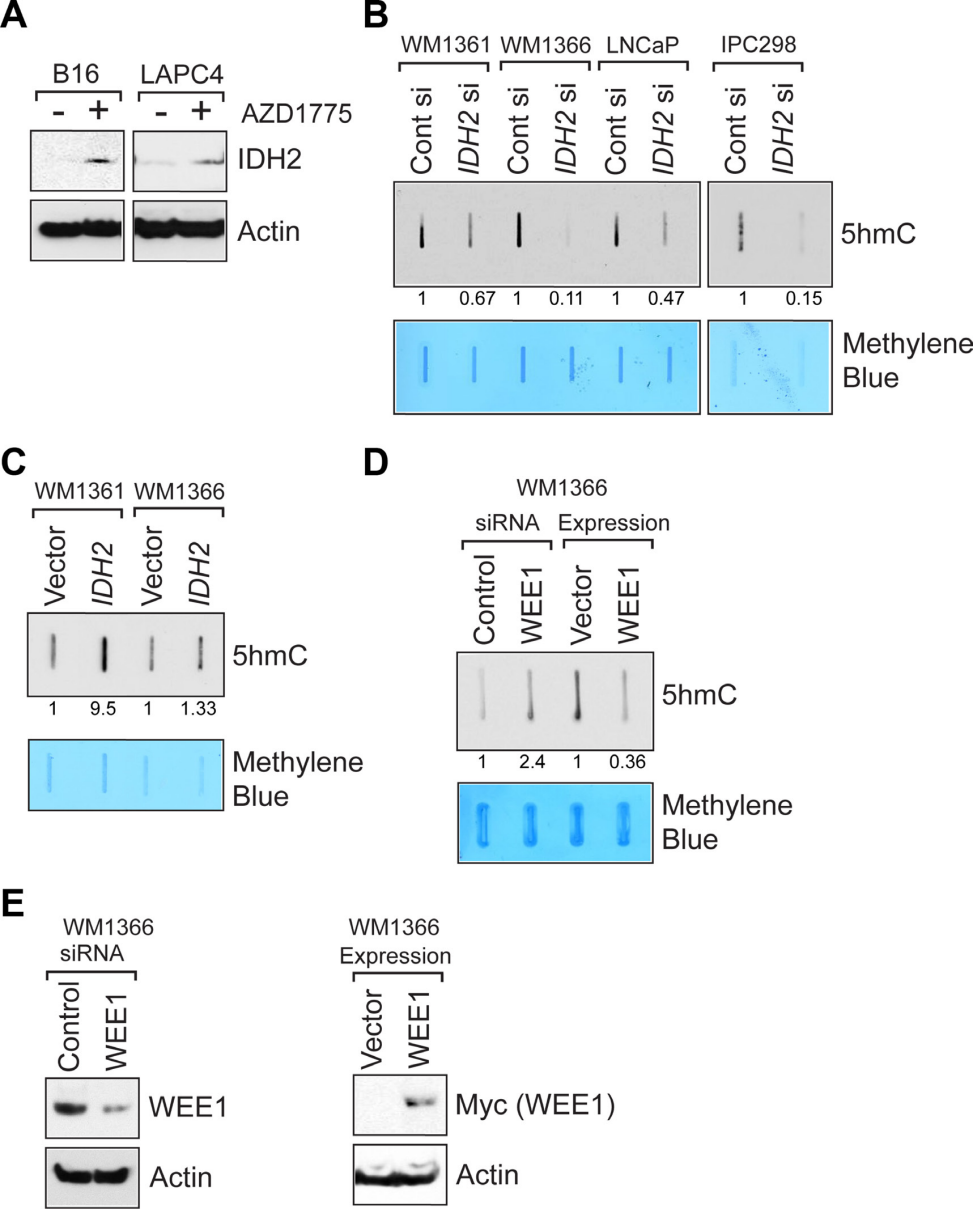
WEE1 negatively regulates *IDH2* protein expression (**A**) Melanoma (B16) and prostate cancer (LAPC4) cells were treated with AZD1775 (2 µM, 24 hr) and lysates were immunoblotted with anti-IDH2 antibodies. Lower panel indicates immunoblotting of lysates with actin antibodies. (**B**) Melanoma (WM1361 and WM1366) and prostate cancer (LNCaP) derived cells were transfected with IDH2 siRNA and total genomic DNA was isolated. Equal amount of DNA was immobilized onto nytran membrane, followed by immunoblotting with 5-hmC antibodies. Numbers shown below top panel indicates relative intensity of the bands compared to untreated controls. Lower panel indicates methylene blue staining of the same blot to confirm equal loading of the DNAs. (**C**) Melanoma cells were transfected with human IDH2 expressing construct and total genomic DNA was isolated. Equal amount of DNA was immobilized onto nytran membrane, followed by immunoblotting with anti-5-hmC antibodies. Numbers shown below top panel indicates relative intensity of the bands compared to untreated controls. Lower panel indicates methylene blue staining of the same blot to confirm equal loading of the DNAs. (**D**) WM1366 cells were transfected with either WEE1 or control siRNA or human WEE1 (Myc-tagged) expressing construct. Total genomic DNA was isolated. Equal amount of DNA was immobilized onto nytran membrane, followed by immunoblotting with anti-5-hmC antibodies. Numbers shown below top panel indicates relative intensity of the bands compared to untreated controls. Lower panel indicates methylene blue staining of the same blot to confirm equal loading of the DNAs. (**E**) WM1366 cells were transfected with either WEE1 or control siRNA or human WEE1 (Myc-tagged) expressing construct. Lysates were immunoblotted with indicated antibodies.

To assess whether the upregulation of IDH2 gene transcription by WEE1 inhibition also reflects in IDH2 protein levels, melanoma (B16) and prostate (LAPC4) cells were treated with AZD1775 and lysates were immunoblotted with IDH2 antibodies. A distinct increase in the IDH2 protein expression was observed upon WEE1 inhibition (Figure 4A). We also assessed whether WEE1 epigenetically regulates *IDH1* gene expression by performing qRT-PCR. AZD1775 treatment did not affect IDH1 mRNA expression (Supplementary Figure 2).

Loss of IDH2 function has previously been linked to decreased 5-hmC levels in human tumors [26–30]. Ease of transfection of WM1361, WM1366, LNCaP and IPC298 has made us use of these four cell lines to transfect with IDH2 siRNA and detect 5hmC, as shown in Figure 4B–4E. To validate loss of IDH2 lead to downregulation of 5-hmC levels, human cancer cells were transfected with *IDH2* siRNA and DNA was isolated followed by immunoblotting. A significant decrease in global 5-hmC levels was observed upon *IDH2* knockdown (Figure 4B). Conversely, overexpression of *IDH2* led to an increase in 5-hmC levels compared to vector control (Figure 4C).

We also assessed the effect of WEE1 loss on 5-hmC levels. siRNA mediated downregulation of WEE1 lead to a significant increase in 5-hmC levels, while overexpression of WEE1 resulted in a significant decrease in 5-hmC levels (Figure 4D and 4E). Taken together, these data suggests that WEE1/pY37-H2B signaling has an important role to play in regulating *IDH2* expression in multiple models.

### 5-hmC loss in cancer cells can be reversed by WEE1 inhibition

**Figure 5:**
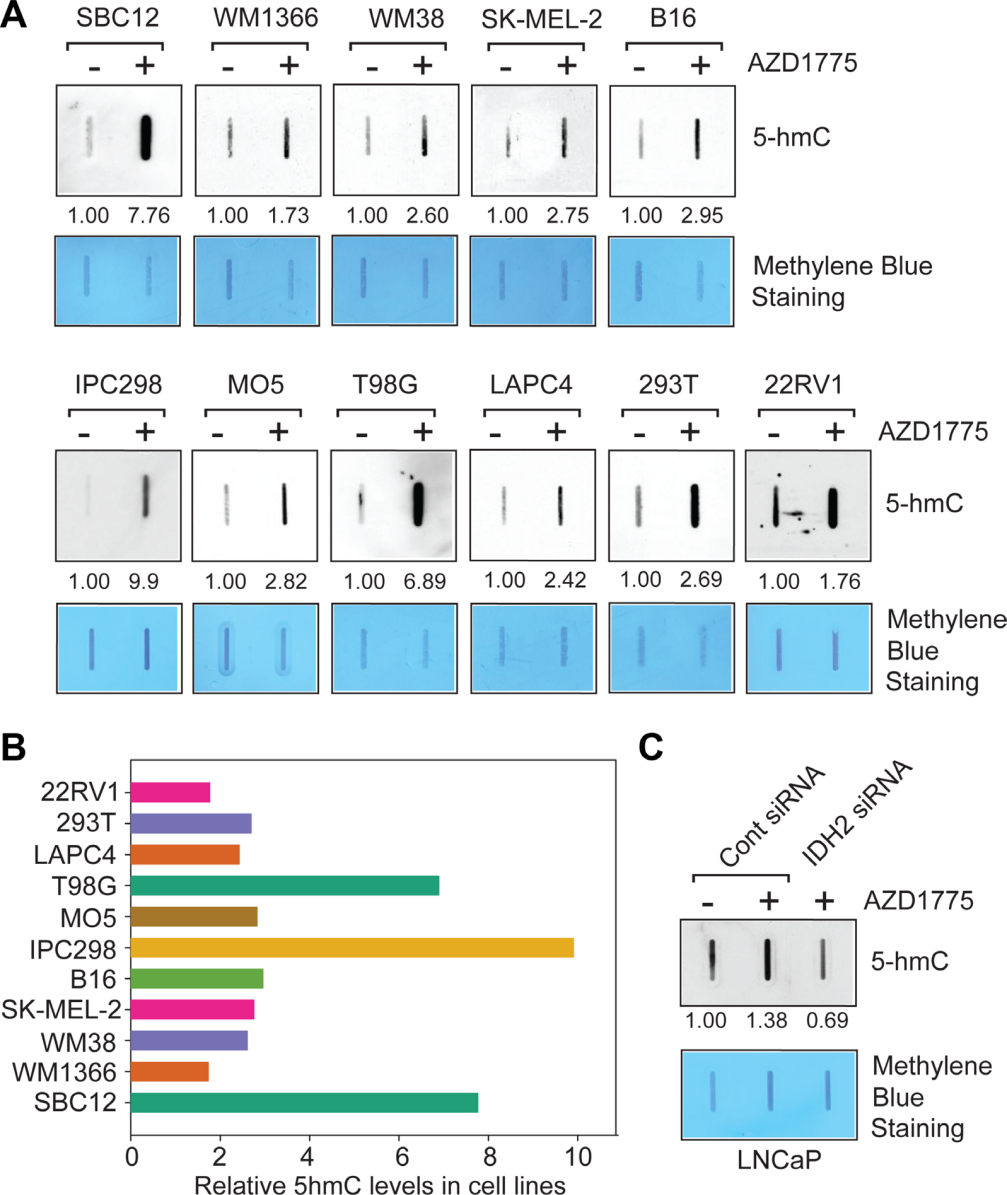
Inhibition of WEE1 kinase activity causes upregulation of 5-hmC levels (**A**) Melanoma, GBM and prostate cancer derived cells were treated with AZD1775 (1 µM, 24 hr) and total genomic DNA was prepared. Equal amount of DNA was immobilized onto nytran membrane, followed by immunoblotting with 5-hmC antibodies. Numbers shown below top panel indicates intensity of the bands compared to untreated controls. Loading of equal amount of DNAs was confirmed by methylene blue staining (lower panels). (**B**) Relative 5-hmC levels in AZD1775 treated cells are shown in comparison to untreated cells. (**C**) LNCaP cells were transfected with IDH2 or control siRNA. 24 hour post transfection, cells were treated with AZD1775 (1 µM, 24 hr) and total genomic DNA was isolated. Equal amount of DNA was immobilized onto nytran membrane, followed by immunoblotting with 5-hmC antibodies. Numbers shown below top panel indicates intensity of the bands.

To determine whether WEE1 mediated *IDH2* transcriptional suppression also impacts 5-hmC levels in different cancer models, melanoma (SBC12, WM1366, WM38, SK-MEL-2, B16, IPC298 and MO5), GBM (T98G) and prostate cancer cells (LAPC4 and 22RV1) were treated with AZD1775 and total genomic DNA was extracted to analyze the 5-hmC status. Genomic DNA was denatured and then blotted onto nitrocellulose membrane, followed by immunoblotting with the 5-hmC antibodies. A significant increase in 5-hmC levels occurred in AZD1775 treated cancer cell lines (Figure 5A). The blot was also stained with methylene blue to confirm equal amounts of blotted DNAs (Figure 5A). The blots were quantitated which revealed that most cell lines exhibited about 2–9 fold increase in 5-hmC levels upon WEE1 inhibition (Figure 5B).

To assess whether the global increase in 5-hmC levels upon WEE1 inhibition can be suppressed by forced *IDH2* knock-down, we transfected LNCaP cells with *IDH2* siRNA and compared with pharmacological blockade following treatment with AZD1775. A significant decrease in global 5-hmC levels was seen upon *IDH2* knock-down in LNCaP cells that was not upregulated in response to AZD1775 treatment (Figure 5C). Taken together with the observed increase in *IDH2* expression, shown in Figure 3, these data indicate that WEE1 mediated deposition of pY37-H2B marks in *IDH2* gene results in transcriptional silencing, causing a significant decrease in global 5-hmC levels.

### GBMs display elevated WEE1 and a significant decrease in *IDH2* mRNA expression profiles

**Figure 6:**
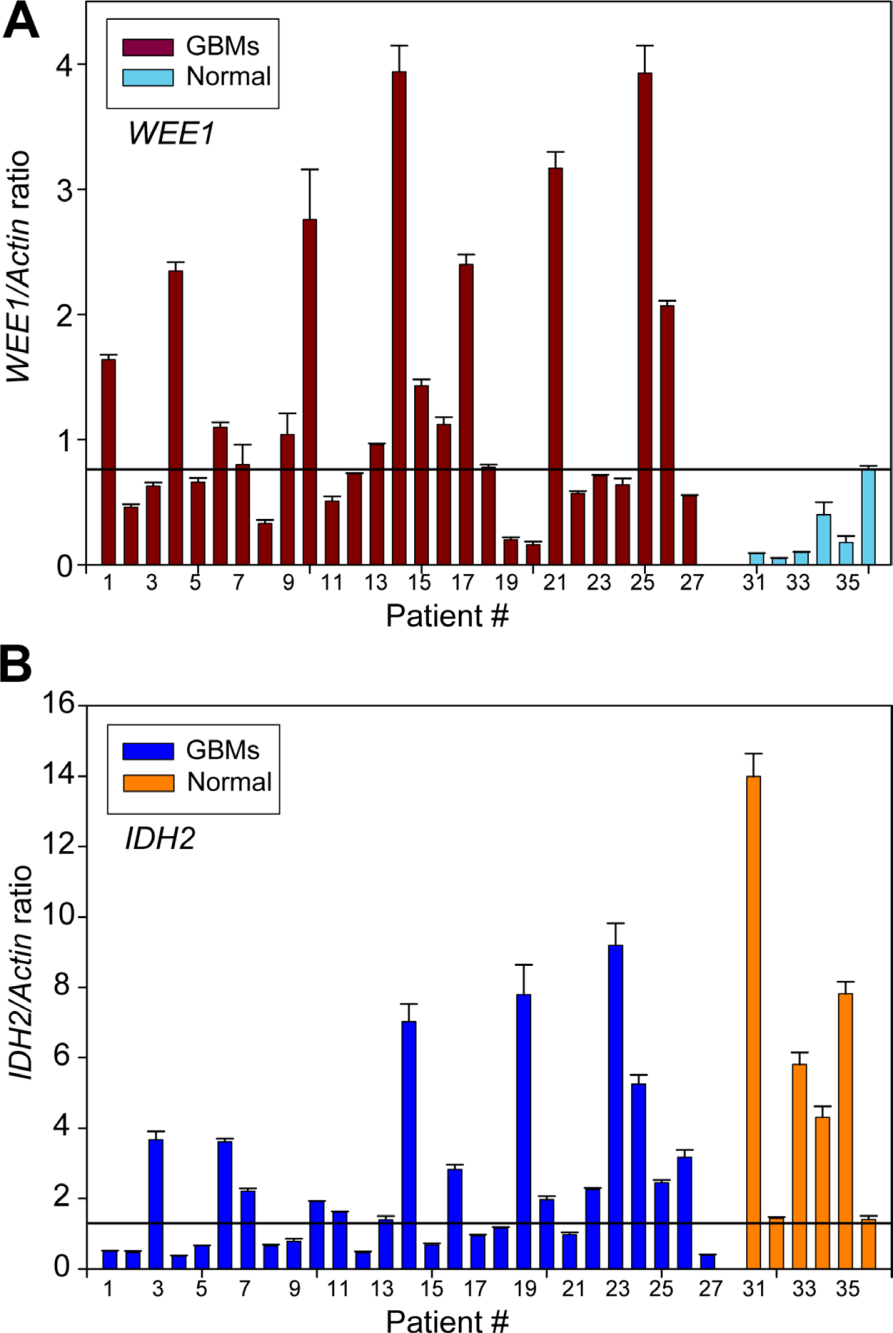
Upregulation of WEE1 and downregulation of *IDH2* mRNA expression profiles in GBM patients Quantitative reverse transcription polymerase chain reaction (qRT–PCR) analysis of *WEE1* (**A**) and *IDH2* (**B**) and actin mRNAs in GBM tumors and normal brain samples. Data represent mean ± standard error of the mean (s.e.m.) (*n* = 3).

Based on our finding that WEE1 epigenetically regulates the expression of chromatin modifying gene *IDH2*, we envisaged that alterations in WEE1 regulatory control could significantly disrupt the epigenetic landscape of GBMs, where increased WEE1 expression has been noted. To interrogate this hypothesis further, we analyzed 27 primary grade IV glioblastomas and 6 normal brain samples. All tumors were microdissected and a pathologist validated each of the tissue samples prior to its usage. Total RNA was prepared, followed by qRT-PCR using *WEE1*, *IDH2* and Actin specific primers. A significant increase in WEE1 mRNA levels is observed in GBM tumors compared to the normal human brain samples (Figure 6A). In contrast to WEE1, a significant decrease in *IDH2* mRNA levels was apparent in GBMs as compared to normal brain samples (Figure 6B).

To identify patients that exhibited a characteristic WEE1/IDH2 signature, the threshold for each variable was selected to maximize the sensitivity (ratio of positives to GBMs) when the specificity (ratio of negatives to normal samples) is set to 1. GBM patients whose WEE1/Actin and IDH2/Actin ratios are >0.774 and <1.27, respectively, are likely to have highly active/elevated WEE1/IDH2 signaling. There were 7 such patients (patient #1, 4, 9, 15, 17, 18 and 21) that fit in this category, indicating that about 26% (7 out of 27) of the GBM patients exhibit elevated WEE1/IDH2 signaling (90% CI: 0.128–0.432). Patient #14 appears to be an outlier, wherein we observed high levels of WEE1 and IDH2 expression. In contrast, none of the normal brain samples exhibited >0.774 and <1.27 ratios for WEE1/Actin and IDH2/Actin, respectively.

### Metastatic melanomas display a significant decrease in *IDH2* mRNA expression

**Figure 7:**
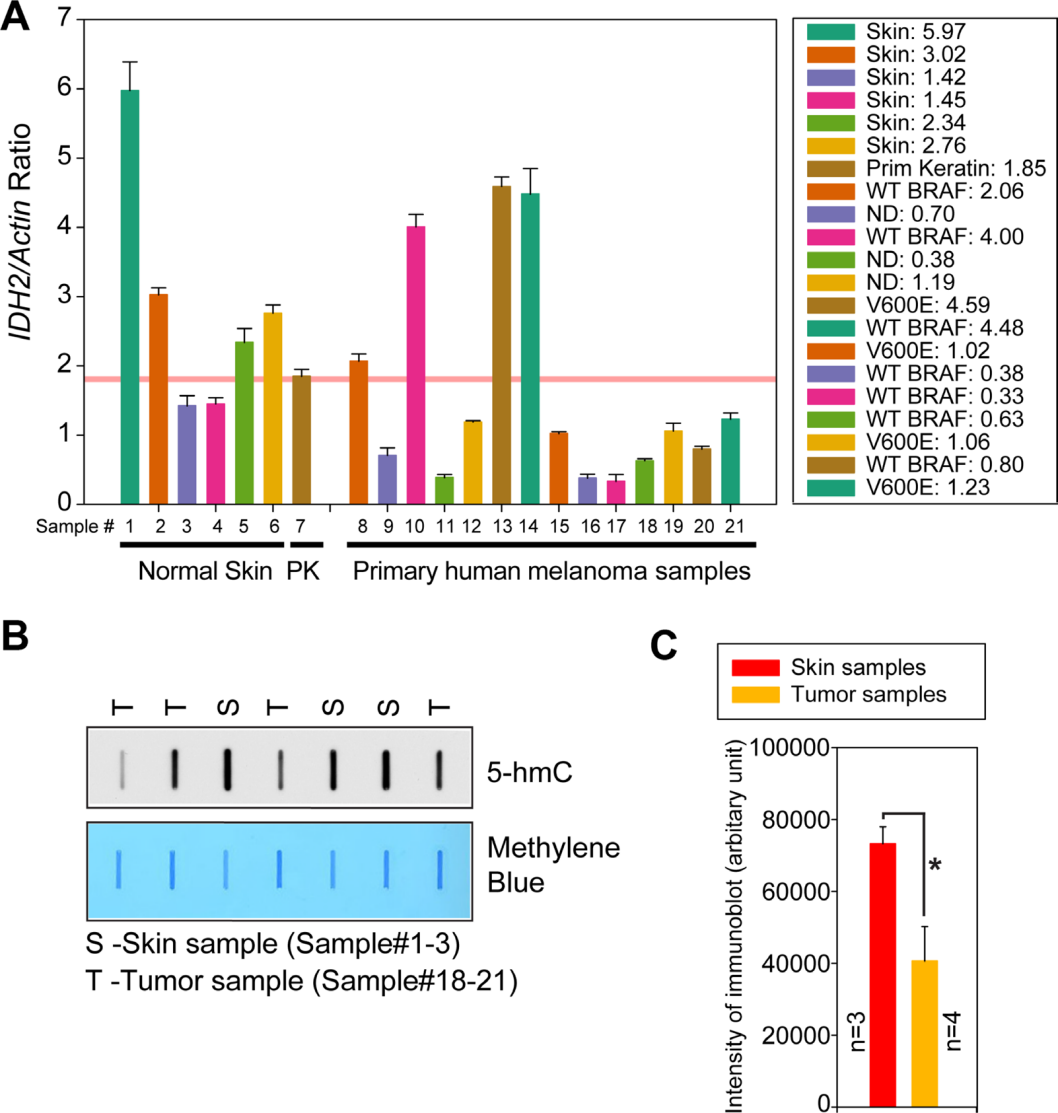
Downregulation of *IDH2* mRNA expression in melanoma patient samples (**A**) Quantitative reverse transcription polymerase chain reaction (qRT–PCR) analysis of *IDH2* and actin RNAs in normal skin samples, primary keratinocytes (PK) and melanoma tumors. Data represent mean ± s.e.m. ND, BRAF status not determined. (**B**) Total genomic DNA was isolated from melanoma and skin samples. Equal amount of DNA was immobilized onto the nytran membrane, followed by immunoblotting with 5-hmC antibodies. Loading of equal amount of DNAs was confirmed by methylene blue staining (lower panels). (**C**) Quantitation of the signal is shown in normal skin (*n* = 3) and tumor sample (*n* = 4).

To interrogate *IDH2* gene expression levels in primary human melanomas total RNA was isolated. As a control RNA was also prepared from 6 normal skin samples and a primary human keratinocyte derived cell line. All tumors were microdissected and a pathologist validated each of the tissue samples prior to its usage. To validate a functional consequence of pY37-H2B deposition at the IDH2 genomic locus, the relative expression of *IDH2* mRNAs was determined based on its ratio with actin. The log-transformation was taken so that the data were normally distributed. A significant decrease in *IDH2* mRNA expression profile was apparent in melanomas as compared to normal skin samples or primary human keratinocyte derived cell line (Figure 7A).

The differences in relative *IDH2* mRNA levels between melanoma and primary human keratinocyte were statistically significant (*p* = 0.048). The *p*-values are two-sided and computed by the two-sample *t*-test. To identify patients that exhibit significant downregulation of *IDH2*, the threshold for each variable was selected to maximize the sensitivity (ratio of positives to melanoma) when the specificity (ratio of negatives to normal samples) is set to 1. These indicated that the melanoma patients whose *IDH2*/Actin ratios are <1.8, are likely to have active/elevated WEE1/pY37-H2B signaling (Figure 7A). There were 10 such patients (patient #9, 11, 12, 15, 16, 17, 18, 19, 20 and 21) that fit in this category, indicating that about 71% (10 of 14) of the melanoma patients exhibit elevated WEE1/pY37-H2B signaling (90% CI: 0.128–0.432). In contrast, two of the 6 normal skin samples (sample 3 and 4) exhibited <1.8 ratios for *IDH2/Actin*. Interestingly, 4 out of 7 (51%) WT BRAF patients exhibited a significant suppression of *IDH2* levels (Figure 7A). In addition to BRAF WT melanomas, *IDH2* levels were also assessed in BRAF V600E mutant melanomas. 3 out 4 (75%) mutant BRAF melanoma samples exhibited a significant downregulation of IDH2. These data open up an intriguing possibility that WEE1 epigenetic signaling is likely to be independent of BRAF mutational status.

To further assess the outcome of downregulation of *IDH2* transcription, 5-hmC levels were examined in skin and melanoma samples. A significant decrease in global 5-hmC levels was observed in melanomas as compared to the skin samples (Figure 7B and 7C).

### Suppression of WEE1 epigenetic activity impedes melanoma xenograft tumor growth

**Figure 8:**
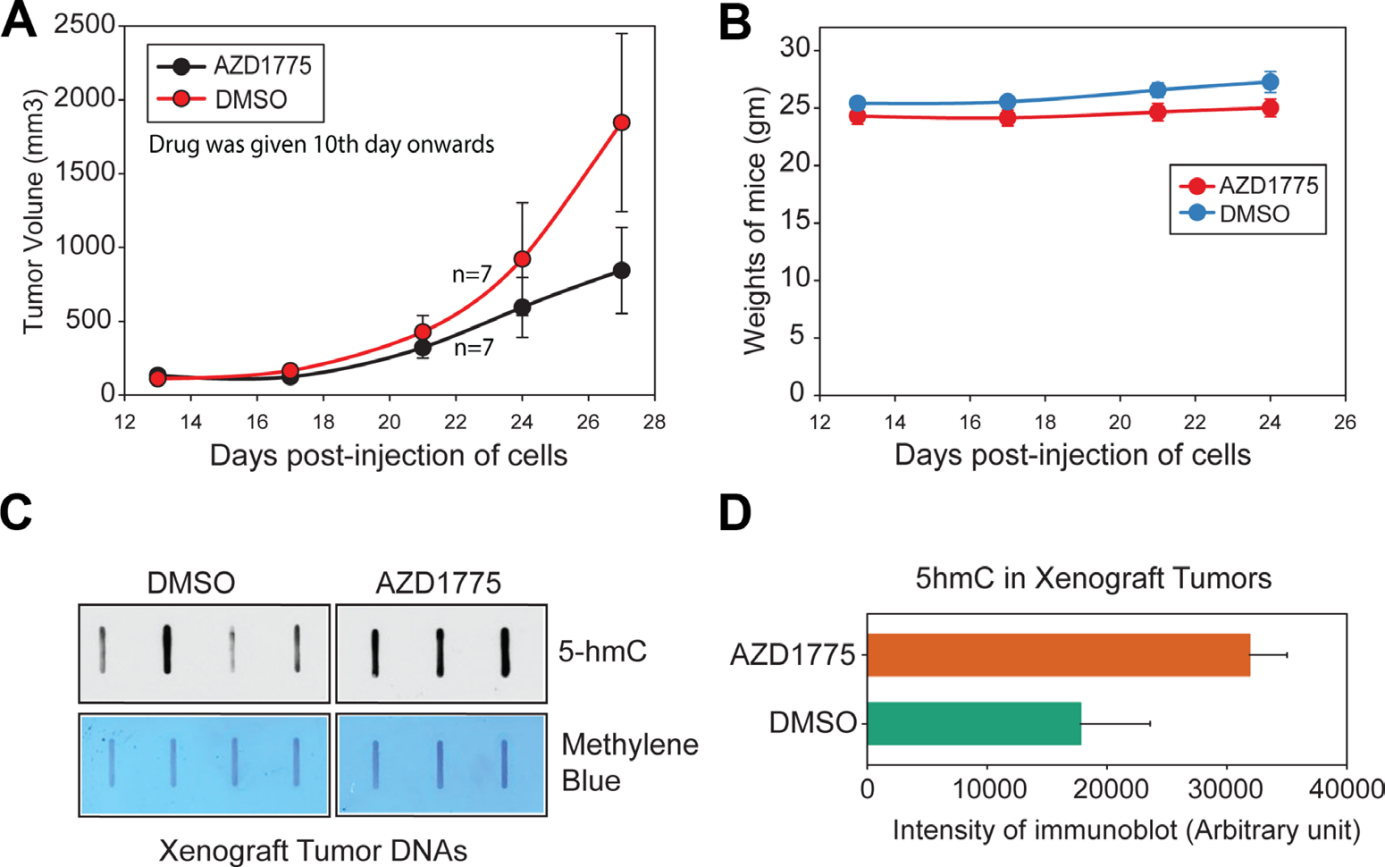
Inhibition of WEE1 activity compromises melanoma xenograft tumor growth (**A**) MO5 melanoma cells were implanted subcutaneously in male B6 mice. When the tumors were palpable mice were given oral gavage with vehicle (10% DMSO in PBS) or AZD1775 (25 mg/kg of body) for 3 days a week (*n* = 7 mice for each treatment). Tumor volumes were measured using calipers. Mean ± s.e.m. (**B**) Weights of the mice are shown. (**C**) DNA was isolated from MO5 xenograft tumors followed by 5-hmc levels assessment by slot blot. (**D**) 5-hmC levels are shown in histogram.

Not only is *WEE1* overexpressed in melanomas, AZD1775, a potent WEE1-specific inhibitor selectively induces apoptosis in WEE1 expressing cancer cell lines [33–35], suggesting that some of the melanomas may be utilizing the WEE1 signaling pathway for survival. To examine whether suppression of WEE1 epigenetic activity impedes melanoma xenograft tumor growth, mouse melanoma cell line MO5 was injected in B6 mice. 10 days post-injection of the cells, when palpable tumors were noticed, mice were given AZD1775 by oral gavage at 25 mg/kg of body weight, three times a week, for 3 weeks. As a control, 10% DMSO (vehicle) was given by oral gavage. Tumor volumes were measured. Decrease in tumor volumes was observed in mice given AZD1775 by oral gavage (Figure 8A). Importantly, this treatment regimen was found to be well tolerated as mice exhibited little weight loss (Figure 8B).

To examine 5-hmC status in xenograft tumors, the tumors were excised and the genomic DNAs was prepared and analyzed. An increase in 5-hmC levels was seen in xenograft tumors isolated from mice that were injected with AZD1775, as compared to DMSO (vehicle) treated mice (Figure 8C and 8D).

## DISCUSSION

**Figure 9:**
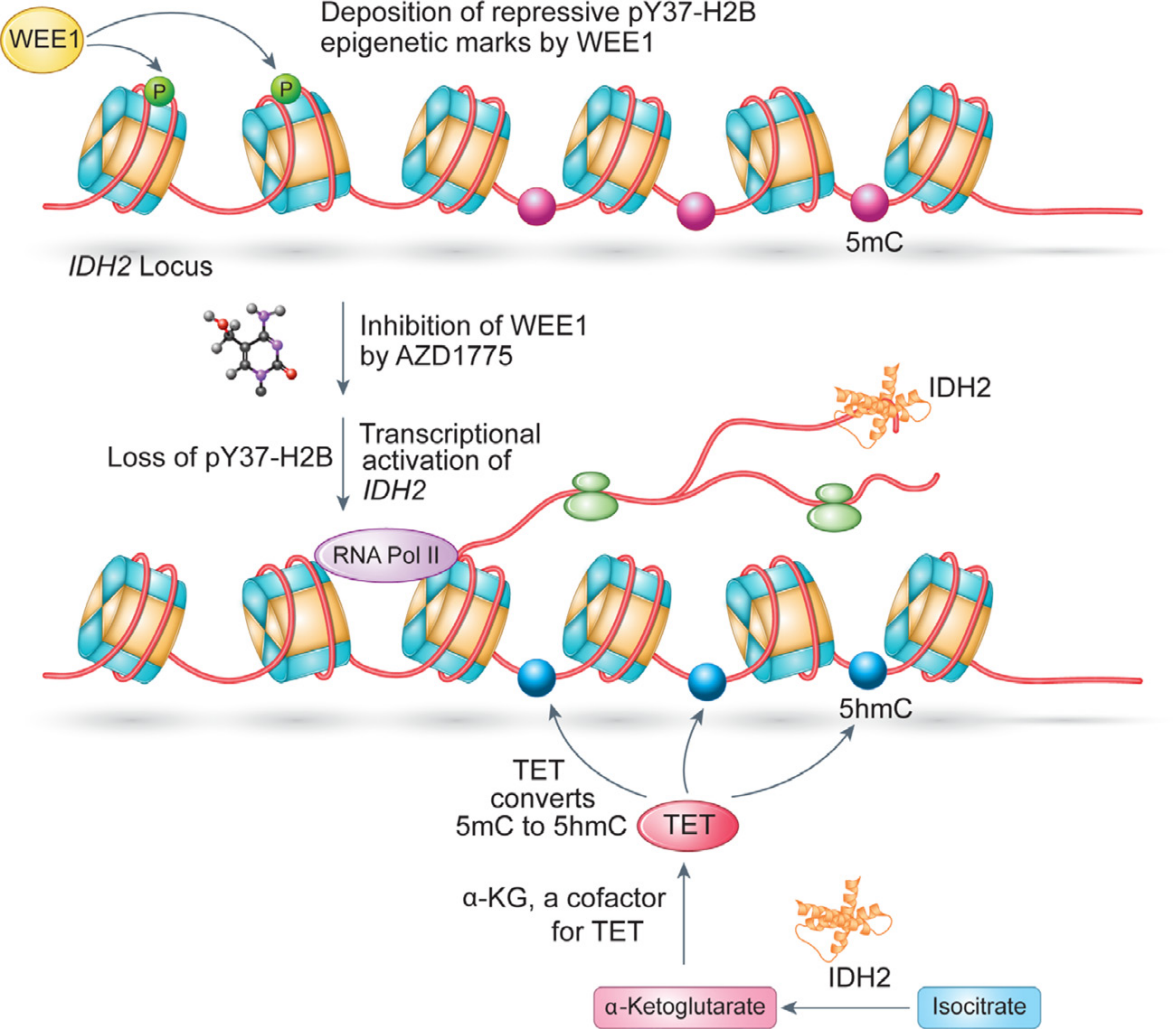
WEE1 pY37-H2B epigenetic signaling axis downregtulates *IDH2* mRNA expression and 5-hmC levels in cancer cell; a model WEE1 overexpressing cancer cells deposits pY37-H2B repressive marks to suppress the expression of *IDH2* transcription which is reflected in a significant loss of global 5-hmC levels that promotes gene expression programs that favor tumor growth. Inhibition of WEE1 by AZD1775 led to the loss of pY37-H2B which in turn restored 5-hmC levels, compromising cancer cell proliferation.

The coordinated control of epigenetic signaling networks ensures proper gene expression programs that are necessary for cellular homeostasis. Although, histone tyrosine phosphorylation is relatively new and understudied epigenetic modification, it has been shown to be involved in cellular processes of high importance. For example, pY37-H2B epigenetic marks were involved in regulating global histone synthesis [7], while pY88-H4 marks regulated androgen receptor expression in the absence of its ligand [14, 36]. Surprisingly, we observed that an epigenetic modification e.g. histone modification regulates another epigenetic modification, a DNA methylation, indirectly via modulating a metabolic and tumor suppressor gene, *IDH2*. These results suggested that the cancer cells could benefit enormously if they were to hijack the WEE1/pY37-H2B epigenetic signaling to regulate DNA methylation in cells. To address this supposition, we examined the mRNA levels of WEE1, IDH2 and Actin (control) in melanoma and GBM tumors and observed that these patients exhibited elevated WEE1 mRNA expression and a significant down regulation of IDH2 transcription (Figure 6). Moreover, ChIP-PCR revealed that pY37-H2B marks were deposited within the *IDH2* coding region indicating a chromatin control of WEE1 transcription (Figure 2). Conversely, inhibition of WEE1 with the specific inhibitor, AZD1775 increased *IDH2* transcript levels and restored 5-hmC levels (Figures 3–5). This is an important finding, as reversal of the 5-hmC levels by WEE1 inhibitor (or any other inhibitor) has not been previously demonstrated. Given the emergence of loss of *IDH2* as an important event in various malignancies, these results suggest that cancer cells overexpressing WEE1 may epigenetically suppress the expression of *IDH2* gene to prevent the formation of 5-hmC (Figure 9). Resulting changes in its cognate epigenetic landscape, may promote gene expression programs that favor tumor growth.

BRAF mutations, particularly at codon 600, are found in almost 50% of the cutaneous melanomas [37], which has led to the successful treatment using BRAF-selective inhibitors, vemurafenib, and dabrafenib, resulting in a prolongation of progression-free and overall survival. Unfortunately, limited treatment options are available for the remaining 50% of the melanoma patients whose tumors are wild-type for BRAF (WT BRAF), with a median overall survival of less than one year [38]. Recent success with PD-1 and PD-L1 antibodies has been encouraging, however further work needs to be undertaken to investigate the possibility of stratifying patients to predict whether or not they will respond to PD-1 therapy. Given the high prevalence of WT BRAF in melanomas, we reasoned that melanomas with wild-type BRAF may exhibit a dependence on WEE1 epigenetic pathway activation for maintenance of the transformed phenotype. Interestingly, the five melanoma cell lines WM1963, IPC298, WM1396, WM3918, SK-MEL-2 that do not possess V600E mutation responded well to the WEE1 inhibitor, increasing *IDH2* mRNA and 5-hmC levels. Further, MO5, a mouse melanoma cell line with WT BRAF too responded to AZD1775, showing tumor regression (Figure 8A). Moreover, 4 of 7 melanoma patients with WT BRAF too exhibited significant downregulation of IDH2 levels. Taken together, these data indicate that WEE1 epigenetic inhibitor could have a significant impact on treatment of melanomas lacking V600 mutation, a critical unmet clinical need.

*IDH1* and *IDH2* mutations have been identified in ∼15–20% of AML and glioma patients^33,34^. These mutations are missense changes. As a result of these mutations, the mutant *IDH1* and *IDH2* acquire a novel enzymatic activity, catalyzing the reduction of α-KG to an oncometabolite, 2-hydroxyglutarate (2-HG) [24, 39]. The 2-HG competitively inhibits α-KG-dependent enzymes such as histone demethylases and TET2, which not only resulted in an increased dimethylation of histone H3K9 and H3K79, but also caused a loss of 5hmC levels in the DNA [24, 25]. Both *IDH1* and *IDH2* mutations were identified in ∼15% of gliomas, which were predominantly seen in grade II-III gliomas and secondary glioblastomas [40, 41]. Very few mutations were identified in primary GBMs (about 3–5%) [41, 42]. Interestingly, in spite of the general absence of *IDH1* and *IDH2* mutations in primary GBMs, 5-hmC levels were significantly reduced in GBMs [27, 32]. Importantly, the loss of 5-hmC in brain cancer did not exhibit any correlation with *IDH1* or *IDH2* mutations [27]. Similar 5-hmC depletion is reported in other cancers, including lung cancer, where its levels do not correlate with *IDH* mutations [32]. Furthermore, mutations in *IDH1* or *IDH2* genes have not been identified in melanomas, but still, virtually all malignant melanoma samples exhibited either a partial or complete loss of 5-hmC [26]. Collectively, these data indicate that *IDH1/2* mutations leading to an increased methylation of H3K9 and H3K79 and decreased 5-hmC may occur in a context-dependent manner in a subset of Acute Myeloid Leukemias, oligodendrogliomas and astrocytomas. Absence of *IDH1* and *IDH2* mutations in the melanoma (or low occurrence in GBMs) indicates that these mutations are unlikely to be the major determinants promoting a loss of 5-hmC in melanomas, primary GBMs and prostate cancers.

Overall, the inhibition of WEE1 erased pY37-H2B repressive marks, causing a marked increase in the levels of *IDH2*, thus reversing the ‛loss of the 5-hmC’ phenotype (Figure 9). Future development of WEE1 ‛epigenetic inhibitor’ could facilitate specific targeting of many of WT BRAF melanomas, GBMs, triple negative breast cancers (TNBCs) and castration resistant prostate (CRPCs), which currently have a few therapeutic options.

## MATERIALS AND METHODS

### Ethics statement

Mice breeding and colony maintenance was performed according to IACUC protocols approved in writing by University of South Florida (USF) Division of Research Integrity and Compliance. We used the human tissues for our study, which were obtained from Moffitt Cancer Center under SRC/IRB approved protocol, MCC#15375, IRB Study #106509 (PI: Sarnaik). The samples were obtained in accordance with the approved guidelines. All experimental protocols were approved by Moffitt Cancer Center & USF SRC/IRB committee. A written informed consent was obtained from all subjects prior to collection of samples.

### Cell lines, antibodies, inhibitors

LNCaP and VCaP cells were obtained from ATCC. GBM cell lines were provided by Peter A. Forsyth. Melanoma cell lines were obtained from laboratory of Keiran Smalley at the Moffitt Cancer Center. IDH2 and WEE1 siRNA were obtained from Dharmacon. For most experiments, cells were treated with 1 µM AZD1775 for 24 hr. WEE1, Actin and H2B antibodies were purchased from Santacruz. 5hmC antibodies were purchased from Active Motif. The small molecule inhibitor, AZD1775 was purchased from Selleck Chemicals. H2B Y37-specific antibodies have been previously described [7].

### Mouse xenograft studies

All animal experimentation were performed using the standards for humane care in accordance with the NIH Guide for the Care and Use of Laboratory Animals (IACUC Protocol # 4151R). 3 × 10^5^ MO5 cells were suspended in 200 µl of PBS with 50% matrigel (BD Biosciences) and were implanted subcutaneously into the dorsal flank of six week old male B6 mice. Once the tumors reached approximately 100 mm^3^ in size (about 10 days), mice were given AZD1775 (or 10% DMSO in PBS as vehicle) at the concentrations 25 mg/kg of body weight, three times a week, for 3 weeks. Tumor volumes were measured twice weekly using calipers. At the end of the study, all mice were humanely euthanized, tumors extracted and weighed. Additionally, spleen, liver and spleen were harvested and stained with H&E to determine effect of inhibitor at the organ level.

### Cell synchronization using a double thyimidine block

MEFs were grown to 60–70% confluency. Thymidine is added at a final concentration of 2 mM and incubated for 17 hours. Cells were washed and fresh serum containing media was added. After 10 hours, thymidine was added again and cells were incubated for 17 hours; cells were washed, replaced with serum containing media and time points were collected.

### Chromatin immunoprecipitation (ChIP) and ChIP-sequencing

Cells (5 × 10^7^ cells) were either untreated or treated with AZD1775. Cells were harvested, fixed in p-formaldehyde and nuclei were prepared. Purified nuclei were resuspended in RLB buffer [43] and sonicated for 25 seconds. The soluble chromatin was incubated overnight at 4°C with antibodies and protein-G or A magnetic beads. The soluble chromatin was processed in the same way without immunoprecipitation and termed input DNA. The amount of immunoprecipitated DNA was determined by real-time PCR. The complexes were washed with ChIP buffer 1 and 2 (Active Motif), eluted with elution buffer and subjected to proteinase-K treatment. ChIP DNA was subjected to proteinase-K treatment and was purified using PCR DNA purification column.

### Quantitative RT-PCR and ChIP-PCR

All RT reactions were done at the same time so that the same reactions could be used for all gene studies. For the construction of standard curves, serial dilutions of pooled sample RNA were used (50, 10, 2, 0.4, 0.08, and 0.016 ng) per reverse transcriptase reaction. One “no RNA” control and one “no Reverse Transcriptase” control were included for the standard curve. Three reactions were performed for each sample: 10 ng, 0.8 ng, and a NoRT (10 ng) control. Real-time quantitative PCR analyses were performed using the ABI PRISM 7900HT Sequence Detection System (Applied Biosystems). All standards, the no template control (H_2_O), the No RNA control, the no Reverse Transcriptase control, and the no amplification control (Bluescript plasmid) were tested in six wells per gene (2 wells/plate × 3 plates/gene). All samples were tested in triplicate wells each for the 10 ng and 0.8 ng concentrations. The no RT controls were tested in duplicate wells. PCR was carried out with SYBR Green PCR Master Mix (Applied Biosystems) using 2 µl of cDNA (or ChIP DNA) and the primers in a 20-µl final reaction mixture. After 2-min incubation at 50°C, AmpliTaq Gold was activated by 10-min incubation at 95°C, followed by 40 PCR cycles consisting of 15 s of denaturation at 95°C and hybridization of primers for 1 min at 55°C. Dissociation curves were generated for each plate to verify the integrity of the primers. Data were analyzed using SDS software version 2.2.2 and exported into an Excel spreadsheet. The actin data were used for normalizing the gene values; i.e., ng gene/ng actin per well. The primer sequences are shown below.

### Primer sequences for qRT-PCR

**Table:** 

Human IDH2 FP	AGATGGCAGTGGTGTCAAGGAG
Human IDH2 RP	CTGGATGGCATACTGGAAGCAG
Mouse IDH2 FP	GGCTGTCAAGTGTGCCACAATC
Mouse IDH2 RP	TTGGCTCTCTGAAGACGGTTCC
Human ACTIN FP	CACCATTGGCAATGAGCGGTTC
Human ACTIN RP	AGGTCTTTGCGGATGTCCACGT
Human HIST H2AI FP	CGACAACAAGAAGACTCGCATCA
Human HIST H2AI RP	TGTGCGATGGTGACTTTGCCCA

### Primer sequences for ChIP-PCR

**Table:** 

Mouse IDH2 ChIP 3	TAGACGTGAA GGCGCAACTCCGGACAAAAT
Mouse IDH2 ChIP 4	TTC CGT GGCAAC AGGCATCAAAAGAGGCCA
Human IDH2 ChIP 7	TAAGATCTGGTATGAGCACCGGCTCATTGA
Human IDH2 ChIP 8	GACCAACAGTCCACCCCCACAGGGAGCAGC

### RNA isolation from human tumors and cell lines

RNAs from normal skin samples, primary human melanomas, primary human keratinocytes prostate cancer cell lines and glioblastomas were isolated using the Trizol method followed by purification with the Qiagen RNAeasy plus kit that included the genomic DNA elimination step. All the tumors were microdissected and pathologist validated each of the tissue samples prior to its usage. RNA from cell lines was isolated using RNeasy plus kit (Qiagen) that included the genomic DNA elimination step.

### Genomic DNA isolation from xenograft tumors and cell lines

Total genomic DNAs from mouse xenograft tumors were isolated using ZR Genomic DNA Tissue MicroPrep (Zymoresearch, CA). Genomic DNA was isolated from cell lines using Quick gDNA MicroPrep (Zymoresearch, CA).

### 5-hmC analysis by slot blot

1 ug of the genomic DNA was treated with 5 uM NaOH and heated to 95°C for 5 min to denature the DNA. DNA was snap chilled, mixed with 100 uM Ammonium acetate and 0.4 ug of the DNA was blotted onto nitrocellulose membrane. DNA was UV cross-linked and immunoblotted with anti-5-hmC antibody (Active Motif).

### Flow cytometry

Melanoma cell lines were treated or untreated with WEE1 inhibitor AZD1775 (3 uM) overnight for 24 h with vehicle or AZD1775 (Selleck chemicals) and harvested by gentle trypsinization. Cells were stored in 100 μl of sodium citrate buffer. Cells were trypsinized in 450 μl of trypsin solution at room temperature for 10 min, incubated in 375 μl of trypsin inhibitor/RNase A (Sigma) solution for 10 min, stained with 250 μl of ice-cold propidium iodide solution for 10 min, and kept in the dark. Samples were analyzed using the Canto flowcytometer; 10,000 events were collected, and cell cycle analysis was carried out using the ModFit program.

## SUPPLEMENTARY MATERIALS FIGURES AND TABLE



## References

[1] Probst AV, Dunleavy E, Almouzni G (2009). Epigenetic inheritance during the cell cycle. Nat Rev Mol Cell Biol.

[2] Schwartzentruber J, Korshunov A, Liu XY, Jones DT, Pfaff E, Jacob K, Sturm D, Fontebasso AM, Quang DA, Tonjes M, Hovestadt V, Albrecht S, Kool M (2012). Driver mutations in histone H3.3 and chromatin remodelling genes in paediatric glioblastoma. Nature.

[3] Wu G, Broniscer A, McEachron TA, Lu C, Paugh BS, Becksfort J, Qu C, Ding L, Huether R, Parker M, Zhang J, Gajjar A, Dyer MA (2012). Somatic histone H3 alterations in pediatric diffuse intrinsic pontine gliomas and non-brainstem glioblastomas. Nat Genet.

[4] Sturm D, Witt H, Hovestadt V, Khuong-Quang DA, Jones DT, Konermann C, Pfaff E, Tonjes M, Sill M, Bender S, Kool M, Zapatka M, Becker N (2012). Hotspot mutations in H3F3A and IDH1 define distinct epigenetic and biological subgroups of glioblastoma. Cancer Cell.

[5] Brower V (2011). Epigenetics: Unravelling the cancer code. Nature.

[6] Burgess RJ, Zhang Z (2013). Histone chaperones in nucleosome assembly and human disease. Nat Struct Mol Biol.

[7] Mahajan K, Fang B, Koomen JM, Mahajan NP (2012). H2B Tyr37 phosphorylation suppresses expression of replication-dependent core histone genes. Nat Struct Mol Biol.

[8] Dawson MA, Bannister AJ, Gottgens B, Foster SD, Bartke T, Green AR, Kouzarides T (2009). JAK2 phosphorylates histone H3Y41 and excludes HP1alpha from chromatin. Nature.

[9] Xiao A, Li H, Shechter D, Ahn SH, Fabrizio LA, Erdjument-Bromage H, Ishibe-Murakami S, Wang B, Tempst P, Hofmann K, Patel DJ, Elledge SJ, Allis CD (2009). WSTF regulates the H2A.X DNA damage response via a novel tyrosine kinase activity. Nature.

[10] Chou RH, Wang YN, Hsieh YH, Li LY, Xia W, Chang WC, Chang LC, Cheng CC, Lai CC, Hsu JL, Chang WJ, Chiang SY, Lee HJ (2014). EGFR modulates DNA synthesis and repair through Tyr phosphorylation of histone H4. Dev Cell.

[11] Singh RK, Kabbaj MH, Paik J, Gunjan A (2009). Histone levels are regulated by phosphorylation and ubiquitylation-dependent proteolysis. Nat Cell Biol.

[12] Singh RK, Gunjan A (2011). Histone tyrosine phosphorylation comes of age. Epigenetics.

[13] Mahajan K, Mahajan NP (2013). WEE1 tyrosine kinase, a novel epigenetic modifier. Trends Genet.

[14] Mahajan K, Malla P, Lawrence HR, Chen Z, Kumar-Sinha C, Malik R, Shukla S, Kim J, Coppola D, Lawrence NJ, Mahajan NP (2017). ACK1/TNK2 Regulates Histone H4 Tyr88-phosphorylation and AR Gene Expression in Castration-Resistant Prostate Cancer. Cancer Cell.

[15] Mir SE, De Witt Hamer PC, Krawczyk PM, Balaj L, Claes A, Niers JM, Van Tilborg AA, Zwinderman AH, Geerts D, Kaspers GJ, Peter Vandertop W, Cloos J, Tannous BA (2010). *In silico* analysis of kinase expression identifies WEE1 as a gatekeeper against mitotic catastrophe in glioblastoma. Cancer Cell.

[16] Wuchty S, Arjona D, Li A, Kotliarov Y, Walling J, Ahn S, Zhang A, Maric D, Anolik R, Zenklusen JC, Fine HA (2011). Prediction of Associations between microRNAs and Gene Expression in Glioma Biology. PLoS One.

[17] Magnussen GI, Holm R, Emilsen E, Rosnes AK, Slipicevic A, Florenes VA (2012). High expression of Wee1 is associated with poor disease-free survival in malignant melanoma: potential for targeted therapy. PLoS One.

[18] Aarts M, Sharpe R, Garcia-Murillas I, Gevensleben H, Hurd MS, Shumway SD, Toniatti C, Ashworth A, Turner NC (2012). Forced mitotic entry of S-phase cells as a therapeutic strategy induced by inhibition of WEE1. Cancer Discov.

[19] Iorns E, Lord CJ, Grigoriadis A, McDonald S, Fenwick K, Mackay A, Mein CA, Natrajan R, Savage K, Tamber N, Reis-Filho JS, Turner NC, Ashworth A (2009). Integrated functional, gene expression and genomic analysis for the identification of cancer targets. PLoS One.

[20] Hirai H, Iwasawa Y, Okada M, Arai T, Nishibata T, Kobayashi M, Kimura T, Kaneko N, Ohtani J, Yamanaka K, Itadani H, Takahashi-Suzuki I, Fukasawa K (2009). Small-molecule inhibition of Wee1 kinase by MK-1775 selectively sensitizes p53-deficient tumor cells to DNA-damaging agents. Mol Cancer Ther.

[21] Hirai H, Arai T, Okada M, Nishibata T, Kobayashi M, Sakai N, Imagaki K, Ohtani J, Sakai T, Yoshizumi T, Mizuarai S, Iwasawa Y, Kotani H (2010). MK-1775, a small molecule Wee1 inhibitor, enhances anti-tumor efficacy of various DNA-damaging agents, including 5-fluorouracil. Cancer Biol Ther.

[22] Bridges KA, Hirai H, Buser CA, Brooks C, Liu H, Buchholz TA, Molkentine JM, Mason KA, Meyn RE (2011). MK-1775, a novel Wee1 kinase inhibitor, radiosensitizes p53-defective human tumor cells. Clin Cancer Res.

[23] Mollapour M, Tsutsumi S, Donnelly AC, Beebe K, Tokita MJ, Lee MJ, Lee S, Morra G, Bourboulia D, Scroggins BT, Colombo G, Blagg BS, Panaretou B (2010). Swe1Wee1-dependent tyrosine phosphorylation of Hsp90 regulates distinct facets of chaperone function. Mol Cell.

[24] Xu W, Yang H, Liu Y, Yang Y, Wang P, Kim SH, Ito S, Yang C, Wang P, Xiao MT, Liu LX, Jiang WQ, Liu J (2011). Oncometabolite 2-hydroxyglutarate is a competitive inhibitor of alpha-ketoglutarate-dependent dioxygenases. Cancer Cell.

[25] Reitman ZJ, Yan H (2010). Isocitrate dehydrogenase 1 and 2 mutations in cancer: alterations at a crossroads of cellular metabolism. J Natl Cancer Inst.

[26] Lian CG, Xu Y, Ceol C, Wu F, Larson A, Dresser K, Xu W, Tan L, Hu Y, Zhan Q, Lee CW, Hu D, Lian BQ (2012). Loss of 5-hydroxymethylcytosine is an epigenetic hallmark of melanoma. Cell.

[27] Orr BA, Haffner MC, Nelson WG, Yegnasubramanian S, Eberhart CG (2012). Decreased 5-hydroxymethylcytosine is associated with neural progenitor phenotype in normal brain and shorter survival in malignant glioma. PloS One.

[28] Krell D, Assoku M, Galloway M, Mulholland P, Tomlinson I, Bardella C (2011). Screen for IDH1, IDH2, IDH3, D2HGDH and L2HGDH mutations in glioblastoma. PloS One.

[29] Munari E, Chaux A, Vaghasia AM, Taheri D, Karram S, Bezerra SM, Gonzalez Roibon N, Nelson WG, Yegnasubramanian S, Netto GJ, Haffner MC (2016). Global 5-Hydroxymethylcytosine Levels Are Profoundly Reduced in Multiple Genitourinary Malignancies. PloS One.

[30] Yang H, Liu Y, Bai F, Zhang JY, Ma SH, Liu J, Xu ZD, Zhu HG, Ling ZQ, Ye D, Guan KL, Xiong Y (2013). Tumor development is associated with decrease of TET gene expression and 5-methylcytosine hydroxylation. Oncogene.

[31] Haffner MC, Chaux A, Meeker AK, Esopi DM, Gerber J, Pellakuru LG, Toubaji A, Argani P, Iacobuzio-Donahue C, Nelson WG, Netto GJ, De Marzo AM, Yegnasubramanian S (2011). Global 5-hydroxymethylcytosine content is significantly reduced in tissue stem/progenitor cell compartments and in human cancers. Oncotarget.

[32] Jin SG, Jiang Y, Qiu R, Rauch TA, Wang Y, Schackert G, Krex D, Lu Q, Pfeifer GP (2011). 5-Hydroxymethylcytosine is strongly depleted in human cancers but its levels do not correlate with IDH1 mutations. Cancer Res.

[33] Leijen S, van Geel RM, Pavlick AC, Tibes R, Rosen L, Razak AR, Lam R, Demuth T, Rose S, Lee MA, Freshwater T, Shumway S, Liang LW (2016). Phase I Study Evaluating WEE1 Inhibitor AZD1775 As Monotherapy and in Combination With Gemcitabine, Cisplatin, or Carboplatin in Patients With Advanced Solid Tumors. J Clin Oncol.

[34] Sarcar B, Kahali S, Prabhu AH, Shumway SD, Xu Y, Demuth T, Chinnaiyan P (2011). Targeting radiation-induced G checkpoint activation with the Wee-1 inhibitor MK-1775 in glioblastoma cell lines. Mol Cancer Ther.

[35] De Witt Hamer PC, Mir SE, Noske D, Van Noorden CJ, Wurdinger T (2011). WEE1 kinase targeting combined with DNA-damaging cancer therapy catalyzes mitotic catastrophe. Clin Cancer Res.

[36] Mahajan KN, Mahajan NP (2015). ACK1/TNK2 tyrosine kinase: molecular signaling and evolving role in cancers. Oncogene.

[37] Davies H, Bignell GR, Cox C, Stephens P, Edkins S, Clegg S, Teague J, Woffendin H, Garnett MJ, Bottomley W, Davis N, Dicks E, Ewing R (2002). Mutations of the BRAF gene in human cancer. Nature.

[38] Tsao H, Atkins MB, Sober AJ (2004). Management of cutaneous melanoma. N Engl J Med.

[39] Dang L, White DW, Gross S, Bennett BD, Bittinger MA, Driggers EM, Fantin VR, Jang HG, Jin S, Keenan MC, Marks KM, Prins RM, Ward PS (2009). Cancer-associated IDH1 mutations produce 2-hydroxyglutarate. Nature.

[40] Parsons DW, Jones S, Zhang X, Lin JC, Leary RJ, Angenendt P, Mankoo P, Carter H, Siu IM, Gallia GL, Olivi A, McLendon R, Rasheed BA (2008). An integrated genomic analysis of human glioblastoma multiforme. Science.

[41] Yan H, Parsons DW, Jin G, McLendon R, Rasheed BA, Yuan W, Kos I, Batinic-Haberle I, Jones S, Riggins GJ, Friedman H, Friedman A, Reardon D (2009). IDH1 and IDH2 mutations in gliomas. N Engl J Med.

[42] Ichimura K, Pearson DM, Kocialkowski S, Backlund LM, Chan R, Jones DT, Collins VP (2009). IDH1 mutations are present in the majority of common adult gliomas but rare in primary glioblastomas. Neuro Oncol.

[43] Mahajan K, Coppola D, Challa S, Fang B, Chen YA, Zhu W, Lopez AS, Koomen J, Engelman RW, Rivera C, Muraoka-Cook RS, Cheng JQ, Schonbrunn E (2010). Ack1 mediated AKT/PKB tyrosine 176 phosphorylation regulates its activation. PloS One.

