# Supplementary material for: WEE1 epigenetically modulates 5-hmC levels by pY37-H2B dependent regulation of IDH2 gene expression
Source: Oncotarget. 2017 Nov 10;8(63):106352–68. doi: 10.18632/oncotarget.22374 (PMC5739739; doi:10.18632/oncotarget.22374)
Supplement: Supplementary file 1 [file oncotarget-08-106352-s001.pdf]

WEE1 epigenetically modulates 5-hmC levels by pY37-H2B dependent regulation of *IDH2* gene expression

SUPPLEMENTARY MATERIALS

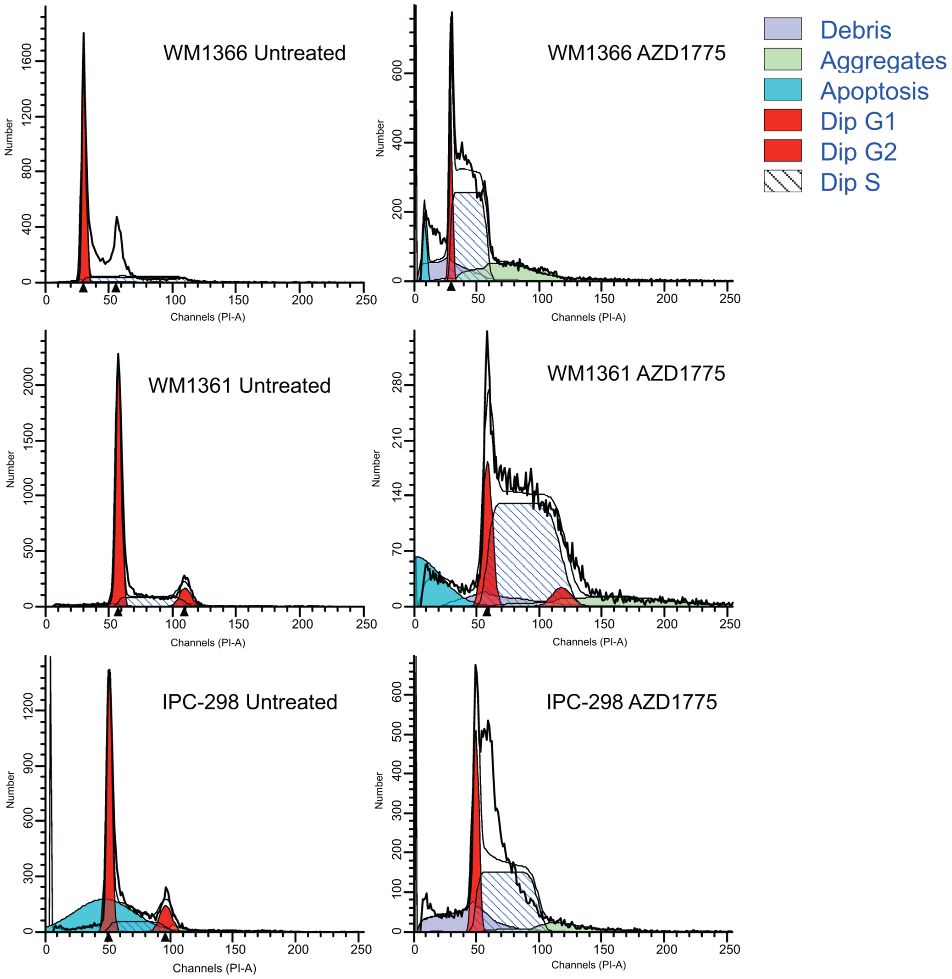

Cells/Condition	Apoptotic/Sub G1	G1 Phase	S Phase	G2/M Phase
WM1366 Untreated	0	67.8	32.2	0
WM1366 AZD1775	5	20.3	79.6	0
WM1361 Untreated	1.3	65.8	24	10
WM1361 AZD1775	13	17.7	77.5	4.6
IPC-298 Untreated	0	67	19.8	13.4
IPC-298 AZD1775	0	29	70.9	0

Supplementary Figure 1: Cell cycle analysis of melanoma cell lines treated with WEE1 inhibitor. Melanoma cell lines were treated or untreated with WEE1 inhibitor AZD1775 (3 uM) overnight. The cells were harvested and were stained with propidium iodide followed by flow cytometry for cell cycle analysis.

Supplementary Table 1: Location of pY37-H2B epigenetic mark depositions in human and mouse genomes

	Start nt	End nt	Location	Chromosome
Mouse	80,114,198	80,114,864	Intron 1–2	7
Human	90,087,067	90,087,236	Exon 7 & part of Intron 7–8	15

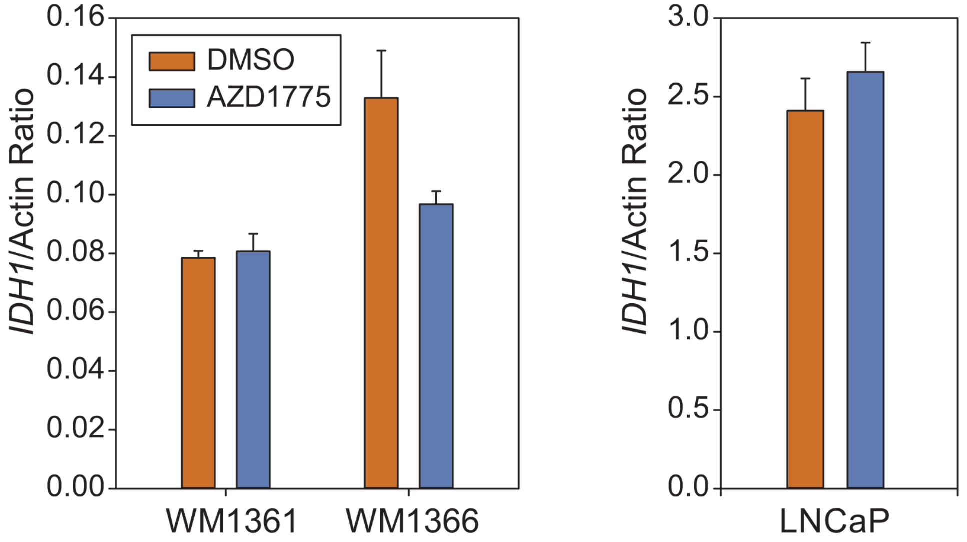

Supplementary Figure 2: Inhibition of WEE1 kinase activity does not affect *IDH1* mRNA expression. Quantitative reverse transcription polymerase chain reaction (qRT–PCR) analysis of *IDH1* mRNA expression in indicated cells treated with AZD1775 (1 µM, 24 hr). Data represent mean ± s.e.m. ($n = 3$).
